# Immunogenic properties of the human gut-associated archaeon *Methanomassiliicoccus luminyensis* and its susceptibility to antimicrobial peptides

**DOI:** 10.1371/journal.pone.0185919

**Published:** 2017-10-05

**Authors:** Corinna Bang, Tim Vierbuchen, Thomas Gutsmann, Holger Heine, Ruth A. Schmitz

**Affiliations:** 1 Institute for General Microbiology, Christian-Albrechts-University Kiel, Kiel, Germany; 2 Division of Innate Immunity, Research Center Borstel, Airway Research Center North, Member of the German Center for Lung Research (DZL), Borstel, Germany; 3 Division of Biophysics, Research Center Borstel, Borstel, Germany; Katholieke Universiteit Leuven Rega Institute for Medical Research, BELGIUM

## Abstract

The methanogenic archaeon *Methanomassiliicoccus luminyensis* strain B10^T^ was isolated from human feces just a few years ago. Due to its remarkable metabolic properties, particularly the degradation of trimethylamines, this strain was supposed to be used as “Archaebiotic” during metabolic disorders of the human intestine. However, there is still no data published regarding adaptations to the natural habitat of *M*. *luminyensis* as it has been shown for the other two reported mucosa-associated methanoarchaea. This study aimed at unraveling susceptibility of *M*. *luminyensis* to antimicrobial peptides as well as its immunogenicity. By using the established microtiter plate assay adapted to the anaerobic growth requirements of methanogenic archaea, we demonstrated that *M*. *luminyensis* is highly sensitive against LL32, a derivative of human cathelicidin (MIC = 2 μM). However, the strain was highly resistant against the porcine lysin NK-2 (MIC = 10 μM) and the synthetic antilipopolysaccharide peptide (Lpep) (MIC>10 μM) and overall differed from the two other methanoarchaea, *Methanobrevibacter smithii* and *Methanosphaera stadtmanae* in respect to AMP sensitivity. Moreover, only weak immunogenic potential of *M*. *luminyensis* was demonstrated using peripheral blood mononuclear cells (PBMCs) and monocyte-derived dendritic cells (moDCs) by determining release of pro-inflammatory cytokines. Overall, our findings clearly demonstrate that the archaeal gut inhabitant *M*. *luminyensis* is susceptible to the release of human-derived antimicrobial peptides and exhibits low immunogenicity towards human immune cells *in vitro*–revealing characteristics of a typical commensal gut microbe.

## Introduction

The human gut microbiota is by far described to be dominated by bacteria [1], although a large number of microeukaryotes, fungi, viruses as well as archaea also form part of it [2]. In contrast to bacteria, fungi and viruses, the immunological impact of archaea on the human immune homeostasis has rarely been described. This underrepresentation is mainly because still no archaeal pathogen is known and the challenges of growing these microorganisms in the laboratory.

The two methanoarchaeal strains *Methanosphaera stadtmanae* and *Methanobrevibacter smithii* were isolated and cultivated from human feces over 30 years ago [3, 4]. Recently, human immune cell activation in response to these two strains was investigated [5, 6] and it was shown that they are prone to the lytic effects of antimicrobial peptides, similarly to those described for bacteria, though they differed in sensitivities [7]. Only recently, several strains from the newly identified methanogenic order Methanomassiliicoccales were also found to inhabit the human intestine with low abundances [8–10] but only the strain *Methanomassiliicoccus luminyensis* strain B10^T^ was cultured and biochemically characterized in 2014 [11]. Importantly, this strain was shown to degrade trimethylamine (TMA) via H_2_-dependent reduction of methyl-compounds in the process of methanogenesis [11]. Thus, the authors hypothesized that human-associated Methanomassiliicoccales strains might be used as probiotics against metabolic disorders associated with TMA produced by gut bacteria [12]. These disorders include trimethylaminuria [13] as well as the development of cardiovascular [14] and chronic kidney disease [15]. However, besides its natural metabolic capacity to diminish TMA very little is known about the functional role of Methanomassiliicoccales within the human intestine and its microbiota. When discussing a potential use of Methanomassiliicoccales as “Archaebiotics” [12], particularly their impact on human immune homeostasis has to be considered.

As mentioned above, the predominant mucosa-associated archaeal strains *M*. *stadtmanae* and *M*. *smithii* appear to have highly different effects on human immune cells, particularly with respect to their overall immunogenicity and sensitivity to mammalian-derived antimicrobial peptides (AMPs) [5, 7]. The corresponding information is still lacking for the new order of Methanomassiliicoccales but it is crucial for a holistic view on its functional role within the human intestine. Therefore, the current study aimed at evaluating those parameters for the cultivable strain *M*. *luminyensis*.

## Materials and methods

### Ethics statement

Approval for these studies was obtained from the Institutional Ethics Committee at the University of Lübeck (Lübeck, Germany; Az. 12-202A) according to the Declaration of Helsinki. All donors gave written informed consent.

### Growth of M. luminyensis

*M*. *luminyensis* (DSM 25720) was obtained from the Deutsche Sammlung von Mikroorganismen und Zellkulturen (DSMZ, Braunschweig, Germany) and was grown at 37°C in 5 or 50 ml minimal medium (Medium 120, DSMZ) under strict anaerobic conditions as described earlier [16]. The medium was reduced with the reductant cysteine (2 mM final concentration) prior to inoculation, 150 mM methanol was added as carbon and energy source and 152 kPa H_2_/CO_2_ (80/20 vol/vol) was used as gas phase. To prevent bacterial contamination the medium was supplemented with 100 μg/ml ampicillin. *M*. *luminyensis* cell numbers were counted in precultures by using a *Thoma* counting chamber and concurrent determination of optical turbidity at 600 nm (OD_600_) during the growth period. For immune cell stimulation, exponentially growing *M*. *luminyensis* cells were harvested at 3,200 x g for 30 min, washed and resuspended in 50 mM Tris-HCl (pH 7.0).

### Antimicrobial peptides (AMPs)

The AMPs tested in this study were a derivative of the human cathelicidin LL37 (LL32) [17], one derivative of porcine NK-lysin (NK2) [18], and a synthetic antilipopolysaccharide peptide Lpep 19–2.5 [19] (all purified from chemical peptide synthesis, and kindly provided by O. Holst, Division of Structural Biochemistry, Research Center Borstel, Borstel, Germany). Peptides were stored in stock solutions at -20°C and diluted in anaerobic Aqua_dest_ prior to use.

### Microtiter plate assay for AMP susceptibility test

The antimicrobial activity of the peptides against *M*. *luminyensis* was determined by growth inhibition in microtiter plates as described earlier [7]. In brief, 2x10^7^ cells from mid-exponential growth phase of precultures were inoculated into 250 μl minimal medium in U-bottom polystyrene microtiter plates (MICROLON**®** - Greiner Bio-One GmbH, Frickenhausen, Germany) and supplemented with different AMP-concentrations. Incubation of these cultures was performed in an anaerobic jar (Schuett-Biotec GmbH, Göttingen, Germany) and 152 kPa H_2_/CO_2_ was continuously supplied during incubation except during monitoring of optical densities in a plate reader that was performed in an anaerobic chamber. After stationary phase was reached, cultures were randomly picked for phase contrast microscopy.

### Cell culture

For peripheral blood mononuclear cell (PBMC) isolation, heparinized blood of donors was prepared by Ficoll (Merck KGaA, Darmstadt, Germany) separation [20]. PBMCs were resuspended at a concentration of 2×10^6^ cells/ml in RPMI medium (Merck KGaA) supplemented with 10% FCS (Merck KGaA) and antibiotics (100 U/ml penicillin and 100 μg/ml streptomycin (both Merck KGaA). Preparation of monocyte-derived dendritic cells was performed by harvesting PBMCs as described above and subsequent isolation of monocytes by counter flow elutriation centrifugation [21]. MoDCs were generated by addition of interleukin 4 (IL-4) and granulocyte-macrophage colony stimulating factor (GM-CSF) to PBMCs in 6-well plates for 7 days as described previously [22], harvested and re-cultured in complemented RPMI medium for stimulation experiments. PBMCs as well as moDCs were grown and incubated in a humidified atmosphere of 5% carbon dioxide at 37°C.

### Cytokine measurements

Cytokine releases of stimulated PBMCs and moDCs were quantified after 20 h by using commercial ELISA Kits (Life Technologies GmbH, Darmstadt, Germany) specific for IL-1β and TNF-α in supernatants.

### Confocal laser scanning microscopy

For confocal laser scanning microscopy 10^5^ moDCs were incubated at 37°C for 2 h on VI channel μ-slides (Ibidi, Martinsried, Germany) before stimulation with 10^7^ Fluorescein isothiocyanate (FITC)-labeled (1 mg/ml, Sigma-Aldrich Chemie GmbH, Hamburg, Germany) methanoarchaeal cells for 16 h. Subsequently, cells were fixed in 3% paraformaldehyde (Biolegend) and labeled with Hoechst 33342 (3 μM, (Life Technologies GmbH)). Images were captured using Leica SP5 confocal microscope (Leica Microsystems GmbH, Wetzlar, Germany) with Leica confocal software.

### Statistical analysis

The data were analyzed for statistical significance using Graph Pad Prism 7.02 software. *P* values ≤ 0.05 were considered to be statistically significant.

## Results

The present study mainly aimed at evaluating the sensitivity of *M*. *luminyensis* to epithelial-derived antimicrobial substances as well as its recognition by humane immune cells with respect to the activation of pro-inflammatory pathways.

### Growth inhibition of *M*. *luminyensis* by AMPs

**Fig 1:**
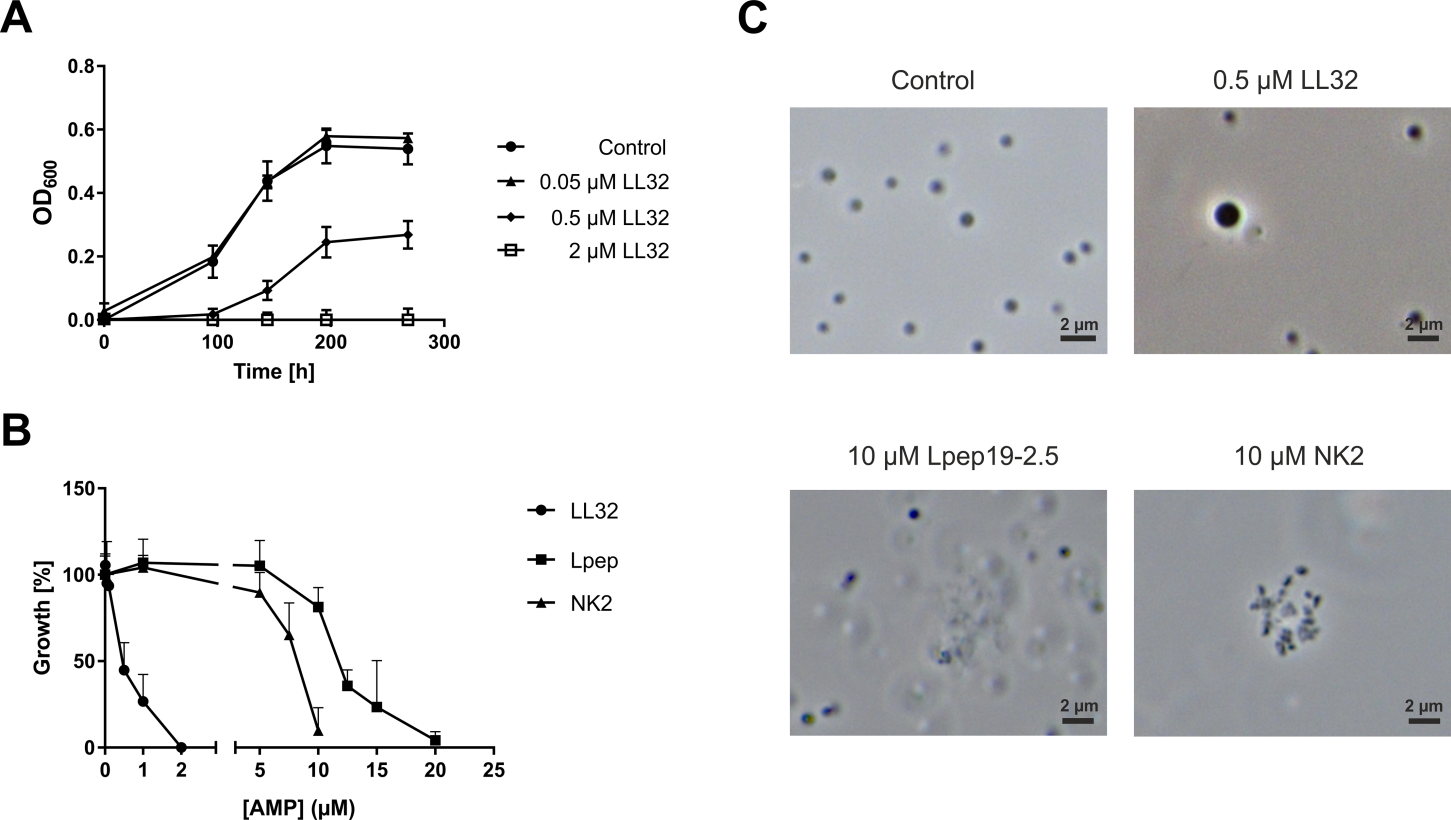
Growth inhibition of *M*. *luminyensis* by various AMPs. **A)** 2x10^7^ cells of *M*. *luminyensis* incubated with depicted concentrations of LL32 at 37°C in 250 μl minimal medium under anaerobic conditions (see Materials and Methods). Turbidity of cultures at 600 nm (OD_600_) was monitored over time. Error bars represent standard deviation of three biological replicates in one experimental setup. **B)** 2x10^7^ cells incubated with various concentrations of the peptides LL32, Lpep 19–2.5 or NK2 at 37°C in 250 μl minimal medium. Turbidity of control cultures at 600 nm (OD_600_) after 200 h of growth was set to 100%. Error bars represent standard deviation of three biological replicates. **C)** Phase-contrast micrographs taken after 200 h of growth with the indicated concentrations of the respective added AMPs.

*M*. *luminyensis* is a coccoid methanoarchaeon that is very small in size (approximately 0.85 μm in diameter) [8]. Earlier studies have shown that this methanoarchaeon can grow on methanol and trimethylamines as carbon sources, but for energy metabolism urgently needs hydrogen as electron donor [8]. To meet the specific growth conditions of *M*. *luminyensis*, the microtiter plate assay that was recently established for anaerobic microorganisms [7] was adjusted. Under optimized growth conditions at 37°C (see Material and Methods) stationary growth phase of *M*. *luminyensis* was reached after 200 h in 250 μl at an optical density of approximately 0.6 (Fig 1A, Control). Cell morphology after growth in microtiter wells was assessed by phase contrast microscopy (Fig 1C, Control). Microscopy also served for exclusion of contaminations within the cultures.

Determination of minimal inhibitory concentrations (MIC) of selected AMPs was performed by adding various concentrations from 5 nM up to 20 μM at the beginning of growth. LL32 is the shortest active unit of human-derived cathelicidin [17], whereas NK2 is a lysin originally isolated from pig [18] and Lpep19.2–5 is a synthetically modified peptide optimized for therapeutic use [23]. Not expected, complete growth inhibition of *M*. *luminyensis* by LL32 was obtained already at low concentrations (2 μM, Tab. 1), whereas inhibition by NK2 as well as Lpep19.2–5 was observed only at relatively high concentrations, 10 μM and 20 μM respectively. In addition, microscopic evaluation during the stationary phase of *M*. *luminyensis* in the presence of AMPs revealed different morphological properties. LL32 resulted in swelling of cells (up to 50% of cell size), which in case of strongly effected cells lead to release of cytoplasmic material from the cells (Fig 1C). In contrast, micrographs of *M*. *luminyensis* after incubation with high concentrations of NK2 and Lpep19.2–5 revealed increased cell coagulation, however only minor effects on overall cell morphology (Fig 1C).

### Immune cell activation by *M*. *luminyensis*

**Fig 2:**
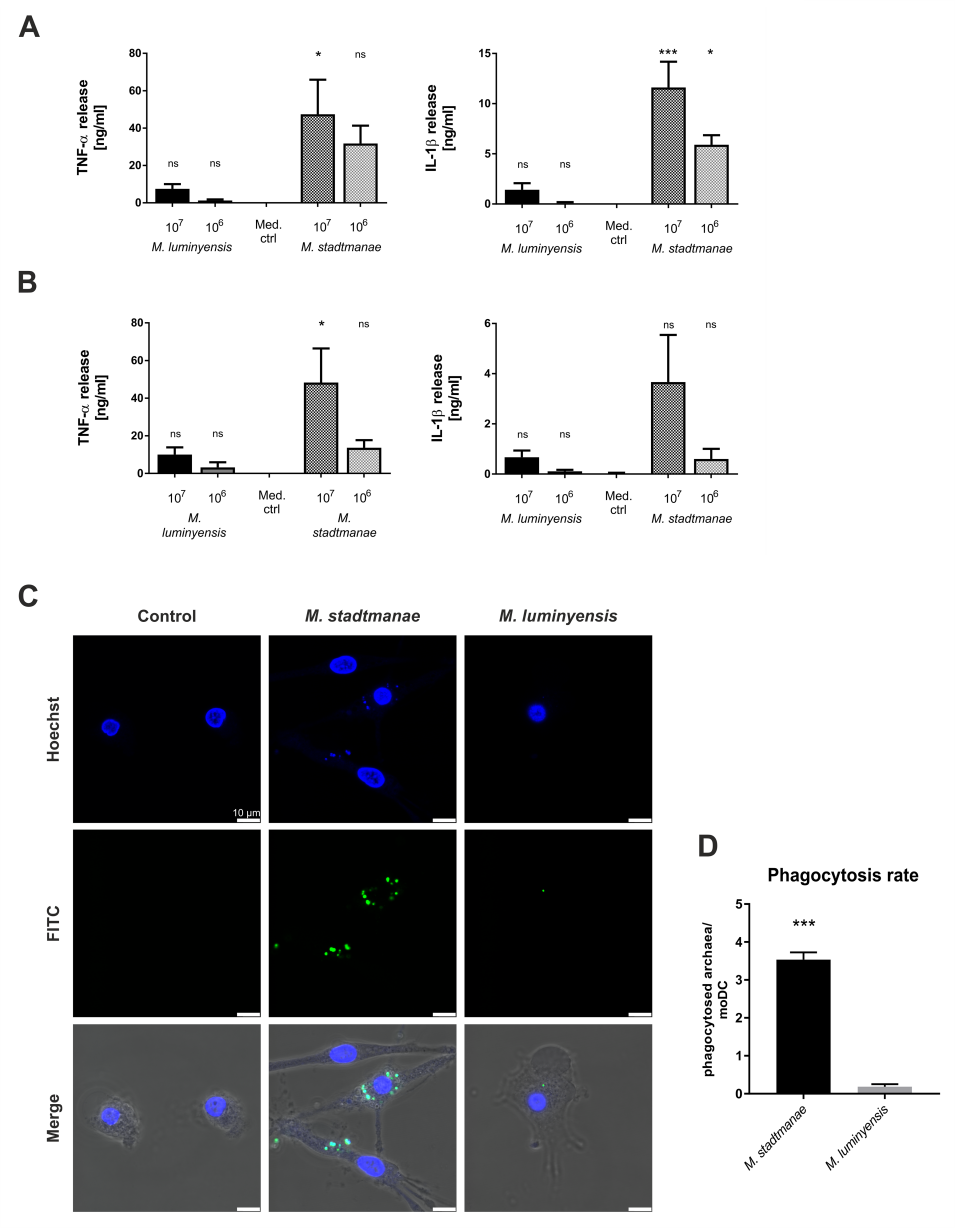
Immune cell activation after stimulation with *M*. *luminyensis*. Cytokine release after stimulation of 1×10^5^ PBMCs **(A)** as well as 1×10^5^ moDCs **(B)** with 1×10^6^ and 1×10^7^ *M*. *luminyensis* or *M*. *stadtmanae* cells for 20 h was quantified using commercial ELISA-Kits. Unstimulated cells (Med. ctrl.) were used as negative controls. Depicted data are means of at least 3 independent biological replicates with their respective standard errors of the mean (SEM). Values are compared to medium control. ns: not significant, * *P*≤0.05, ** *P*≤0.01, *** *P*≤0.001 (one-way ANOVA with Bonferroni post hoc test). **C)** 1×10^5^ moDCs were stimulated with 1×10^7^ FITC-labeled methanoarchaeal cells in VI channel μ-slides for a period of 16 h. After incubation, moDCs were washed, fixed with 3% paraformaldehyde and DNA was labeled with Hoechst 33342. Images were captured using Leica SP5 confocal microscope with Leica confocal software and are representative of the respective samples (three independent biological replicates). **D)** Phagocytosis rate of *M*. *stadtmanae* and *M*. *luminysensis* by moDCs was determined by counting phagocytosed archaeal cells in image sections of three biological replicates (mean of counted moDCs: Medium ctrl = 34, moDCs stimulated with *M*. *stadtmane* = 56, moDCs stimulated with *M*. *luminyensis* = 45). Values are compared to each-other. *** *P*≤0.001 (two-tailed unpaired t-test).

In the current study human PBMCs as well as moDCs were used to evaluate the immunogenic potential of *M*. *luminyensis*, as previously done for the other investigated mucosa-associated methanoarchaea. 1x10^6^ or 1x10^7^ cells of *M*. *luminyensis* were added to preparations of 1x10^5^ PBMCs as well as moDCs. After 20 h of incubation, supernatants were taken and cytokine release of TNF-α and IL-1β was measured by ELISA. *M*. *stadtmanae* served as positive control in these experiments due to its capability to induce very high cytokine releases in primary human immune cells [5, 24]. Compared to medium control, stimulation with *M*. *luminyensis* basically led to the release of pro-inflammatory cytokines TNF-α and IL-1β in both, PBMCs (Fig 2A) and moDCs (Fig 2B), though the amounts were quite low. This effect was observed in a concentration-dependent manner as was also shown for the control stimulus *M*. *stadtmanae* (Fig 2A and 2B, 1x10^6^ and 1x10^7^ cells). In addition to cytokine release, phagosomal uptake by moDCs was evaluated by using CLSM. 1×10^7^ FITC-labeled methanoarchaeal cells were added to 1×10^5^ moDCs seeded in VI channel μ-slides. After 16 h of incubation, DNA was stained with Hoechst 33342 and visualized using confocal microscopy. Whereas the uptake of *M*. *stadtmanae* occurred in high numbers, *M*. *luminyensis* was only rarely found to be phagocytosed by moDCs as depicted in Fig 2C. In addition, the typical change of morphology after activation of immune responses in moDCs such as formation of dendrites was only observed for the positive control *M*. *stadtmanae*.

## Discussion

Although bacteria represent the major microbial part of the human gut ecosystem, recently available molecular tools revealed the constant presence of archaeal species in the gastrointestinal tract [25]. Besides the most prevalent methanoarchaeal strains *M*. *stadtmanae* and *M*. *smithii*, three novel methanoarchaeal species belonging to the Methanomassiliicoccales were isolated and their respective genomes sequenced during the last years [8–10]. Due to their metabolic capability to degrade trimethylamine (TMA) [11] a potential application of naturally occurring Methanomassiliicoccales members as “Archaebiotics” was proposed recently [12]. Since information on the general immunogenic impact of these strains is still missing, the current study mainly aimed to elucidate the molecular cross-talk between components of the human immune system and the cultivable strain *M*. *luminyensis*.

### Susceptibility of *M*. *luminyensis* to AMPs

As a part of the human microflora, methanogenic archaea such as *M*. *stadtmanae*, *M*. *smithii* and *M*. *luminyensis* are exposed to various epithelial as well as immune defense mechanisms that prevent invasion or colonization of organs and maintain homeostasis [26]. One crucial line of epithelial defense is the secretion of AMPs that represent an essential part of immunity within the human intestine [27]. Since AMPs in general exhibit various structural motifs, structurally different AMPs, like a cathelicidin and a NK-lysin derivative as well as a synthetic antilipopolysaccharide peptide (Lpep) were chosen in order to examine the general susceptibility of *M*. *luminyensis*. Moreover, lytic effects of these AMPs on other mucosa-associated methanoarchaeal strains were demonstrated earlier [7]. We demonstrated that *M*. *luminyensis* is indeed susceptible to the human AMP-derivative LL32 in low μM-ranges (see Table 1). In addition, we show that this strain is more resistant to AMPs from other origins such as NK2 and Lpep19-2.5. The latter results resemble those of the other two mucosa-associated methanoarchaeal strains, *M*. *stadtmanae* and *M*. *smithii* summarized in Table 1. When compared to the sensitivity of the other two strains, *M*. *luminyensis* appears to be similarly resistant against the lytic effects of NK2 and Lpep19.2–5 as shown for *M*. *stadtmanae* [7]. However, with respect to this, LL32 showed higher activity against *M*. *luminyensis*. On the other hand, *M*. *luminyensis* is more resistant against all peptides tested than *M*. *smithii*. Concerning the latter it has to be mentioned that based on the small cell size of *M*. *luminyensis*, twice as much cells were used for inoculation (for the reason of growth monitored by turbidity). During earlier studies it was already shown that the cell membrane charge of the tested methanoarchaeal strains, as well as the cell wall structure, strongly influence the interaction and lytic activity of the used cationic charged AMPs [7, 28, 29]. Although the cell wall composition of *M*. *luminyensis* has not yet been fully characterized, transmission electron micrographs indicated a double-layer cell wall with one thin electron-dense layer and one thick transparent layer for this strain [8]. As has been shown for *M*. *stadtmanae* and *M*. *smithii*, *M*. *luminyensis* was found to behave like a Gram-positive in Gram staining assays suggesting that its cell wall might be also composed of pseudomurein [30], however this speculation has to be proven in future studies. With respect to the cell membrane composition, a recent publication demonstrated an unusual membrane lipid composition for *M*. *luminyensis* [31]. Whereas the cell membranes of *M*. *smithii* and *M*. *stadtmanae* are composed of already high amounts of caldarchaeols (13–40%), the cell membrane of *M*. *luminyensis* contains approximately 58% [31]. These caldarchaeols form mono-layer tetraether lipids and thus exhibit a high ordered structure of the membrane. Besides, the cell membrane of *M*. *luminyensis* has been shown to contain only low amounts (~ 33%) of hydrophilic head groups, such as phosphatidylglycerol, and thus probably is less negatively charged, but stabilized by high amounts of lipids with glycosidic head groups [31, 32]. Thus, the differences obtained for *M*. *luminyensis* might be mainly due to its unique cell wall and cell membrane architecture [8, 31]. With respect to the effects of AMPs on structural changes of *M*. *luminyensis*, phase-contrast micrographs revealed swelling of cells after incubation with LL32, but not after treatment with the other peptides. This swelling was most likely due to the osmotic stress after lysis of the cell membrane. During the treatment with NK2 and Lpep19.2–5 *M*. *luminyensis* cells appeared rather to coagulate–an effect that was also observed for *M*. *stadtmanae* during earlier studies and thus coagulation of the cells might resemble a general preventive function against the lytic effects of naturally-occurring AMPs [7]. In conclusion, the results of AMP-treatment regarding growth inhibition and morphological changes of *M*. *luminyensis* revealed that the cell membrane as well as the cell wall appears to be well adapted to mammalian-derived antimicrobial peptides that obtain homeostasis within the intestine.

**Table 1:** Antimicrobial activity of various AMPs against methanogenic archaea. (MIC minimal inhibitory concentration).

	MIC (μM)			Ref.
Strain	LL32	NK2	Lpep 19–2.5	
*M*. *luminyensis*	2	10	> 10	This study
*M*. *stadtmanae*	5	10	> 10	[7]
*M*. *smithii*	1	3	3	[7]

### Immunogenic potential of *M*. *luminyensis*

Previous studies on immune cell activation and responses focusing on cytokine release and CLSM demonstrated that the mucosa-associated methanoarchaeons *M*. *smithii* and *M*. *stadtmanae* led to differential immune cell activation and responses [5, 24]. Based on these results, the question arose, if and how human immune cells respond to *M*. *luminyensis*. Interestingly, we found that stimulation of human immune cells with *M*. *luminyensis* only led to low amounts of the released cytokines TNF-α and IL-1β as it has been demonstrated for the commensal methanoarchaeal strain *M*. *smithii*. In addition, phagocytosis by moDCs as well as the formation of typical dendrites revealing activation was only rarely seen after stimulation with *M*. *luminyensis*, whereas strong activation was obtained after stimulation with *M*. *stadtmanae* as has been observed earlier [33]. These findings suggest that–as it is known for bacteria–diverse (methano)archaeal strains appear to possess structurally different molecular patterns, which serve to a greater or lesser extent as immune activators.

To date, the respective involved human receptor that recognizes archaeal molecular patterns has not been described. However, the pseudomurein-containing cell wall of both, *M*. *stadtmanae* and *M*. *smithii*, is surrounded by a second layer composed of heteropolysaccharides whose structure remains to be elucidated [34]. As mentioned above, the cell wall architecture of *M*. *luminyensis* also appears to be composed of two layers with different structural properties [35]. Since numerous membrane-bound and extracellular receptors are known to be involved in the recognition of bacteria-associated heteropolysaccharides [36], it is conceivable that the outer layer of *M*. *luminyensis* as well as *M*. *smithii* cell walls prevents these strains from phagocytosis by human immune cells. However, in order to support this hypothesis, data on the respective involved human pattern-recognition receptor as well as on the chemical properties of mucosa-associated methanoarchaeal cell wall composition are urgently needed.

Recent studies with particular focus on genomic adaptions demonstrated that *M*. *luminyensis* appears to be the less common gut inhabitant when compared to other members of the Methanomassiliicoccales in the human intestine [37]. In detail, phylogenetic analysis of the so far in human stool detected Methanomassiliicoccales strains revealed that *M*. *luminyensis* is more related to soil and sediment methanogens, whereas the later described strain "Candidatus Methanomethylophilus alvus" was found to be genetically more related to gastrointestinal methanogens [11]. The additional identified strain "*Candidatus* Methanomassiliicoccus intestinalis” is phylogenetically also more related to soil and sediment methanogens, however it has a reduced genome and an up to 20-fold higher prevalence when compared to its closest relative *M*. *luminyensis*. Thus, Borrel and colleagues concluded that *M*. *luminyensis* might not be the best model to study the interactions of the representatives of this archaeal order with their human host [37]. Indeed, it could be speculated that other members of Methanomassiliicoccales might resemble different immunogenetic properties when compared to *M*. *luminyensis*. However, with respect to its metabolic capabilities of TMA depletion, its general appearance in the human intestine and the herein observed overall mild human immune response particularly of this Methanomassiliicoccales strain might be proposed for a potential application as an “Archaebiotic” [12].

## Conclusion

Taken together, this study substantiated adaptation of the intestinal archaeal strain *M*. *luminyensis* to its natural habitat and underlines previous findings on diverse physiological and immunomodulatory roles of other mucosa-associated methanoarchaeal strains in the human intestine.
